# Synthesis and Characterization of a New Peptide Prodrug of Glucosamine with Enhanced Gut Permeability

**DOI:** 10.1371/journal.pone.0126786

**Published:** 2015-05-15

**Authors:** Hamed Gilzad Kohan, Kamaljit Kaur, Fakhreddin Jamali

**Affiliations:** 1 Faculty of Pharmacy and Pharmaceutical Sciences, University of Alberta, Edmonton, Alberta, Canada; 2 Department of Pharmaceutical Sciences, Albany College of Pharmacy and Health Sciences, Albany, New York, United States of America; 3 Chapman University School of Pharmacy (CUSP), Harry and Diane Rinker Health Science Campus, Chapman University, Irvine, California, United States of America; Hungarian Academy of Sciences, HUNGARY

## Abstract

The aim of this study was to synthesize a peptide prodrug of glucosamine (GlcN) with increased gut permeability through the gut *peptide transporter 1* (*PepT1*). Glycine-Valine ester derivative of GlcN (GVG) was synthesised using solid phase synthesis followed by characterization and evaluation of its physicochemical and intestinal stability. In addition, GVG was evaluated for its ability to be biotransformed to GlcN in the liver homogenate. *In vitro* absorption of the new prodrug through everted rat gut was also assessed. GVG demonstrated significant and meaningful increased gut permeability as compared with GlcN. It showed favorable stability in the gut and a quick cleavage to GlcN after exposure to the liver homogenate. In conclusion, a novel prodrug of glucosamine with superior gut permeability compared to GlcN was developed and successfully tested *in vitro*.

## Introduction

Glucosamine (GlcN), a naturally occurring amino-sugar, is a putative disease modifying agent [1] with mild anti-inflammatory properties [2,3] and is commonly administered to treat osteoarthritis (OA) [4]. It has been shown that GlcN significantly suppresses the Interleukin-1 beta (IL-1β) signaling pathway, an essential mediator of the inflammatory responses, by inhibition of NF-κB activation, which in turn leads to a decrease of both inflammatory and degenerative mediators of the disease [1]. Additionally, it has been reported that GlcN inhibits various pro-inflammatory mediators such as, cyclooxygenase-2 (COX-2), nitric oxide (NO), and matrix metalloproteinases (MMP) [5]. Although there are several animal studies indicating the potential anti-inflammatory and disease modifying effects of GlcN [2,3], human clinical trials are controversial [5]. A part of this controversy can be attributed to the limited bioavailability of GlcN [5], which prevents the achievement of therapeutic levels in plasma given the usual administered dosage in humans (1500 mg/day). Indeed, administration of high doses of GlcN in adjuvant model of arthritis in rats can prevent the emergence of inflammation and ameliorate the disease signs in early stages [2,3]. In addition, GlcN is a non-regulated dietary supplement in the US and Canada; hence, the quality of the available products in the market is questionable [6,7]. On the other hand, GlcN sulfate is regulated drug in all Europe and indeed the only clinical trials that had promising results were those with the regulated products [5]. The dosing regimens of GlcN are empirical because of insufficient pharmacologic information. Recently a dose-effect study using a pharmaceutical grade glucosamine formulation revealed that the minimum effective dose to prevent adjuvant arthritis is 40 mg/kg/day in a rat model of adjuvant arthritis [8], which yields much greater concentrations in plasma than the commonly used human doses [9]. In fact only animal [2,3] and human [10] trials that have been carried out with relatively high doses or were associated with high plasma GlcN concentrations have been reported to be effective in controlling inflammatory conditions. Crystalline GlcN sulfate appears to be unstable unless crystalized with stabilizing salt such as NaCl [7]. Hence, the mere size of the available tablets deters patients from taking more than 1500 mg/day to benefit from the treatment, particularly for the elderly patients.

One of the strategies to reduce the required dose of the drugs in order to achieve the desired bioavailability (hence, dose: efficacy ratio), is to synthesize prodrugs [11]. Prodrug design to target the membrane transporters such as *peptide transporter 1* (*PepT1*) has received particular attention in recent decades [11,12]. Several compounds seem to be absorbed efficiently via this path. Peptide transporters are involved in the absorption of a broad range of therapeutic agents, including ACE inhibitors, beta-lactam antibiotics, renin inhibitors, bestatin and valacyclovir, a valine ester prodrug of acyclovir [13–16].

**Fig 1:**
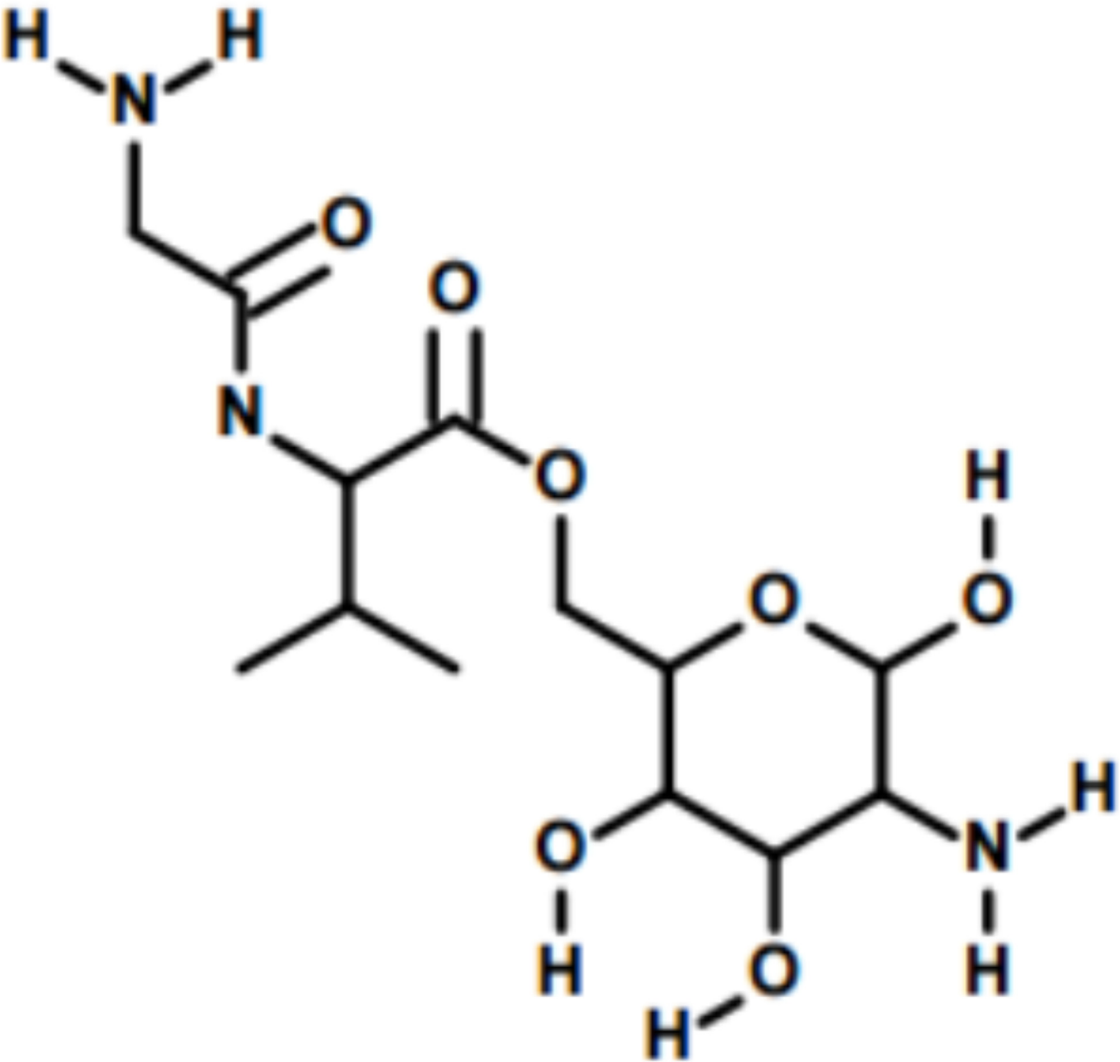
Chemical structure of GVG.

The goal of this study was to synthesize a physico-chemically stable prodrug of GlcN with high stability and permeability through the gut, followed by a rapid release of GlcN in the liver. To this end, a dipeptide-GlcN ester derivative or Glycine-Valine-Glucosamine (GVG, Fig 1) was synthesized using a stepwise solid phase synthesis method on a 2-chlorotrityl chloride resin. The gut permeability of GVG was evaluated using everted jejunum sacks [17]. In addition, in the course of the study, several other mono amino-acid and dipeptide prodrugs of GlcN were synthesized [18]. These other compounds displayed negligible penetration through the gut and/or insufficient conversion to the parent compound (GlcN) in the liver, and were not further investigated.

## Materials and Methods

### Materials

2-chlorotrityl chloride resin, BOP (benzotriazol-1-yl-oxy-tris-(dimethylamino) phosphonium hexafluorophosphate), and HOBt.H2O (hydroxybenzotriazole monohydrate) were purchased from Novabiochem (Merck KGaA, Darmstadt, Germany). Boc-Gly-Val was purchased from Bachem (Bachem Americas, Inc., Torrance, USA). D-Glucosamine HCl, Glycylsarcosine (Gly-Sar), NMM (N-methylmorpholine), mannosamine HCl (MA), amantadine HCl (1-aminoadmantane HCl, ADAM), TEA (triethylamine), TFA (trifluoroacetic acid), DPBS (dulbecco's phosphate buffered saline), *Diaion HP*-*20* resin, and Fmoc-Cl (9-fluorenylmethoxycarbonyl chloride) were purchased from Sigma-Aldrich Canada, Ltd, (Oakville, ON, Canada). HPLC grade acetonitrile (ACN), water, dichloromethane (DCM) and dimethylformamide (DMF) were obtained from Caledon Laboratories Ltd, (ON, Canada). All other chemicals and solvents were commercial products of analytical or HPLC grades.

### Animals

The experimental protocol was approved by the Health Sciences Animal Policy and Welfare Committee of the University of Alberta. Adult male Sprague—Dawley (SD) rats (250–280 g), which were acclimatized in controlled temperature room with a 12:12 h dark/light cycle were used.

### Synthesis and characterization

**Fig 2:**
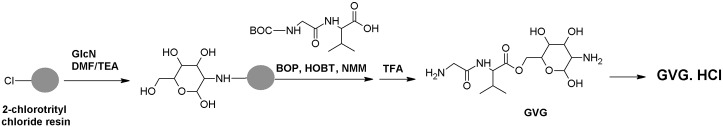
Synthetic scheme for solid-phase synthesis of GVG.

The primary alcohol of GlcN was selected to synthesize the dipeptide GlcN ester derivative. The chemical structure of the Glycine-Valine ester prodrug of GlcN (GVG) is shown in Fig 1. GVG was synthesized using a stepwise synthesis method on a 0.5 mmol scale of 2-chlorotrityl chloride resin (1.2% DVB cross-linked) based on the standard solid-phase peptide synthesis (SPPS) approach as previously described [29], with some modifications (Fig 2). Briefly, GlcN (2 eq) was dissolved in a DMF/TEA (5/1, 10 mL) mixture and added to the pre-swelled resin in DCM. Coupling between the resin and the amine group of the GlcN was achieved by stirring the mixture overnight at room temperature. The resin was then drained to remove all the reagents and solvents, and was washed with DMF and DCM. In the next step, the Boc protected di-peptide (Boc-Glycine-Valine-COOH) (2 eq) was activated by the addition of BOP (2 eq), HOBt (2 eq), and NMM (2 eq) in DMF (6 mL). The activated di-peptide was then added to the resin and the mixture was stirred for several hours at room temperature. The primary alcohol group of GlcN reacted with the activated carboxylate group of the di-peptide. After draining and washing, the final cleavage of the product from the resin was achieved by treating the resin with TFA in DCM (1/1, 6 mL). The Boc removal from the terminal amino group was achieved during the final cleavage step. The resulting solution was dried under vacuum, and then washed with cold ether (4°C) in order to obtain the GVG TFA salt. The trifluoroacetate (TFA) salt of GVG was then converted to hydrochloride salt by treatment with 0.1 M HCl for 45 minutes.

During the synthesis process, formations of the desired derivatives were confirmed by mass spectrometry. The mass analyses were performed using a Waters Micromass ZQ 4000 system equipped with an auto-sampler, pump, and a Waters 2795 separations module (Waters, Milford, MA). The dipeptide GlcN ester derivative was purified using *Diaion HP*-*20* [19] column chromatography. A stepwise procedure was followed for purification of the compound. Glass columns (100 mL) were filled with *Diaion HP-20* resin. *Diaion HP-20* resin was fully hydrated before the purification procedure. The column was loaded with an aqueous solution. The solution (200 mL) was passed through the column followed by 200 mL of water (no leakage of the peptide was observed up to this point). Finally the loaded conjugates were eluted from the resin using an optimum mixture of ACN/and or methanol/water. Throughout the process, a rate of 2 bed volumes per hour was maintained. Finally, the solvents were evaporated to yield the final purified GlcN prodrug. Purified compound was evaluated using mass spectrometry and mass balance study.

The structure of the ester derivative (Gly-Val-COO-GlcN) was further confirmed by 1H and 13C NMR spectroscopy.

### Analytical HPLC assay for GlcN

The HPLC system was comprised of a Shimadzu Prominence system (Mandel Scientific, Guelph, ON Canada) equipped with fluorescence and *photodiode arrays* detectors, and a Phenomenex C18 (100 mm X 4.6 mm, id 3 micron) reversed phase column. The gradient mobile phase consisted of 0.1% acetic acid/ACN at 1 mL/min flow rate. A previously reported assay [30] with some modifications was used to measure GlcN levels in the samples. Briefly, aliquots of the samples (100 μL) were spiked with mannosamine HCl (MA) as the internal standard (IS) and treated with cold ACN (4°C) to precipitate the proteins. They were then, centrifuged and their supernatants were derivatized with Fmoc-Cl (8 mM in ACN) in presence of borate buffer (0.2 M) at 30°C for 30 min. The excess amount of derivatizing agent was removed with ADAM (300 mM in ACN/water 1:1) and 5 μL of the final solution was injected into the HPLC. Standard curves were linear over the concentration range of 0.05 to 20 μg/mL (r^2^ ≥ 0.99, coefficient of variation ≤10%). Mass balance calculation was achieved by determining the amount of GlcN. As each mole of the compounds consists of one mole of GlcN, by measuring the amount of the GlcN formed in each experiment, the amount of the di-peptide-GlcN derivative that was degraded (in the stability tests) or transported (in the gut permeability experiments) could be calculated indirectly.

### Thermal and chemical stability studies

Powder of GVG was transferred into dry transparent glass vials (50 mL) and placed in a 60°C oven for 48 h. At the end of the experiment, a given portion of the compounds was incubated with LiOH, and the GlcN content was measured. The amount of the degraded compound was determined based on a mass balance calculation.

In order to evaluate the effect of pH on the chemical stability of the GlcN conjugate, the prodrug was incubated in an aqueous HCl solution (pH 2) or Krebs-Heneseleit bicarbonate buffer (pH 7.4) at 37°C for 2 h followed by the analytical HPLC assay for GlcN.

### Intestinal and liver homogenate studies

Liver and intestinal homogenate hydrolysis studies were performed according to a previously described method [31] with some modifications. Briefly, rats were euthanized by the method of inhalant anesthetic (isoflurane) followed by cardiac puncture under anesthesia as the subsequent secondary method and their livers and intestines were excised. After being washed with cold dulbecco's phosphate buffered saline (DPBS) (4°C, pH 7.4), specimens were placed in liquid nitrogen for instant freezing and stored at −80°C until further analysis. On the day of experiment, tissues were thawed and homogenized in 10 mL of chilled (4°C) *DPBS* for about 30 s with a tissue homogenizer (Kinematica AG, Littau- Lucerne, Switzerland) in an ice bath. Subsequently, the homogenates were centrifuged at 12,500 G for 25 min at 4°C to remove cellular debris and the supernatants were collected for hydrolysis studies. The protein contents of the samples were determined using a commercially available protein assay kit (Bio-Rad Laboratories, Hercules, CA, USA). Solution of the prodrug (1 mM, 100 uL) were added to 400 μL of the liver or intestinal homogenized supernatants (n = 6) and the mixtures were incubated at 37°C in a shaking water bath. Aliquots of 50 μL were withdrawn at 0, 15, 30, and 60 min, and 50 μL of ice-cold ACN was added in order to precipitate the cellular proteins and stop the reaction. Samples were stored at −80°C until further analysis. Finally, GlcN concentration was determined in all samples and degraded amount of the compounds was determined based on a mass balance calculation. GVG decomposed to GlcN and the di-peptide moiety; by measuring the amount of GlcN released during the experiment (HPLC method), the amount of degraded compounds was calculated indirectly. The measured concentrations from the intestinal homogenate study were plotted against the time points on a *semi-logarithmic graph* and the first order rate constants (k_obs_) and the half-lives (t_1/2_ = 0.693/k_obs_) of the degradation were calculated from the slope of the lines.

### Permeability study through everted rat jejunum sacks

The permeability experiment was performed according to a previously described method [32] with some modifications. Male SD rats (250–280 g) were deprived of food 12 h before the experiment with free access to water. A mid-line incision was made in the abdomen of the anesthetized rats. The intestine was exposed, and six segments (10 cm each) of the jejunum were cut 10 cm after the ligament of Treitz. The segments were immersed immediately into ice cold Krebs-Heneseleit bicarbonate buffer (pH 7.4), and the intestinal contents were removed. Each segment was everted over a glass rod and tied from one side with a silk thread. A silastic catheter (0.58 mm i.d. x 0.965 mm o.d) was inserted into the other side and tied. The segments were filled with 2 ml of the buffer. 30 ml of freshly prepared solution of the prodrug or GlcN (1.5 mM) in the buffer was added to perfusion apparatus vessels (n = 6), and a single everted jejunum sack was immersed in each vessel in order to introduce the compound solutions to the mucosal (outer surface of the everted gut) side. The solutions were oxygenated by a mixture of O_2_/CO_2_ (95/5) during the experiment, and temperature was kept at 37°C. Serial samples (0.5 mL) were collected from both mucosal and serosal sides at 0, 15, 30, and 60 min. The withdrawn volume was replaced with an equal volume of the Krebs buffer. Samples were kept frozen at -20°C until the day of analysis. On the day of the analysis, samples were thawed and incubated with lithium hydroxide (LiOH, 1 N) for 2 h in order to cleave the ester bond and yield the parent drug (GlcN) and the di-peptide moiety. The amount of GlcN transferred into the serosal site and remained in the mucosal site was measured using the analytical HPLC assay for GlcN; this amount represented the amount of the prodrug moved from the mucosal side toward the serosal side. Cumulative amount of prodrug inside the jejunum sacks (the serosal side) were calculated and compared with the values for GlcN. In addition, percent average ratio of GlcN concentration inside/outside the jejunum sacks (n = 6) after 60 min incubation with GVG was calculated and compared to that of GlcN.

Furthermore, permeability inhibition test for GVG in the presence and absence of a specific *PepT1* substrate, Gly-Sar, was carried out. Rats’ jejunum sacks were pre-incubated either with Gly-Sar (10 mM) or Krebs-Heneseleit bicarbonate buffer for 5 min, and a sufficient amount of GVG was then added to the solution to make a final concentration of 1.5 mM. Aliquots of the samples (0.5 mL) were collected from both the mucosal and the serosal sides at 60 min. Samples were incubated with LiOH to cleave the ester bond in order to yield the parent drug (GlcN). Finally the GlcN concentration inside and outside of the jejunum sacks was measured using the analytical HPLC assay for GlcN, and the percent ratio of GlcN concentration inside/outside the jejunum sacks was calculated.

### Statistical analysis

Data are presented as mean ±S.D. Differences among the cumulative amount of GlcN (μg) inside the jejunum sacks, and the percent average ratio of GlcN concentration inside /outside the jejunum sacks after 60 min was determined utilizing a one-way ANOVA, followed by Bonferroni post-test. Statistical analyses were carried out using Prism software (GraphPad Software Inc., San Diego, CA, USA) at *P < 0*.*05*.

## Results

### Synthesis and characterization

Dipeptide-GlcN prodrug, GVG, was designed to target the *PepT1* for enhancing the bioavailability of the parent drug, GlcN. The synthesis of GVG was achieved using solid-phase synthesis on 2-chlorotrityl chloride resin (Fig 2).

**Fig 3:**
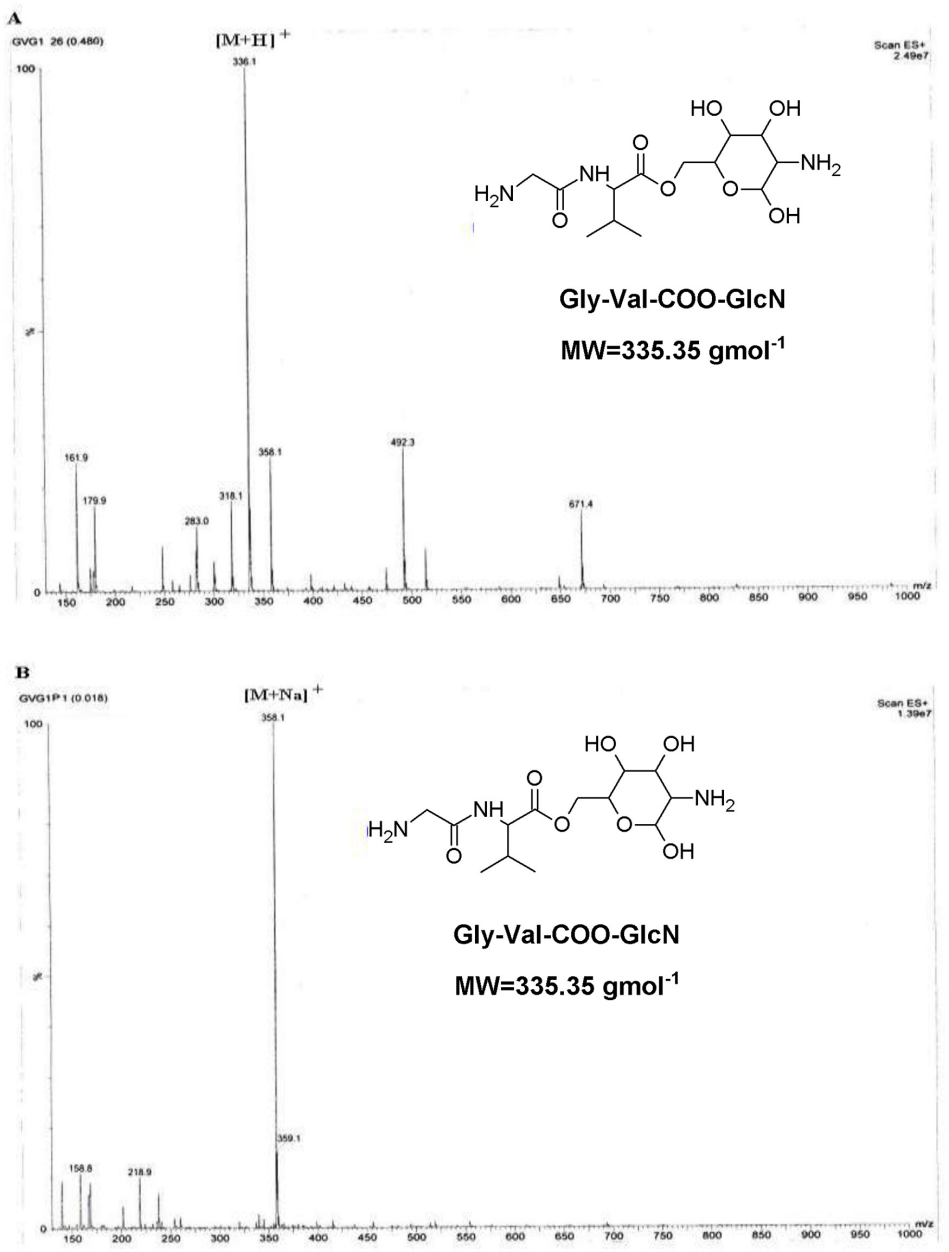
Mass spectra of the Gly-Val-COO-GlcN (GVG) conjugate before (A) and after (B) purification using *Diaion HP*-*20* resin (MW = 335.357).

First, GlcN was coupled to the resin followed by addition of the Boc-protected dipeptide using BOP and HOBt as coupling agents. Finally, GVG was cleaved from the resin with concomitant removal of the BOC group using trifluoroacetic acid. The trifluoroacetate salt of GVG was converted to hydrochloride salt by treatment with dilute HCl. Crude compound was purified using *Diaion HP*-*20* [19] column chromatography to give final pure crystalline solid compound (hydrochloride salt) with >98% purity and 67.8% yield. The identity of the compound was determined using mass spectrometry. Fig 3 presents the mass spectra of the GVG conjugate before **(A)** and after **(B)** purification using *Diaion HP*-*20* resin.

**Fig 4:**
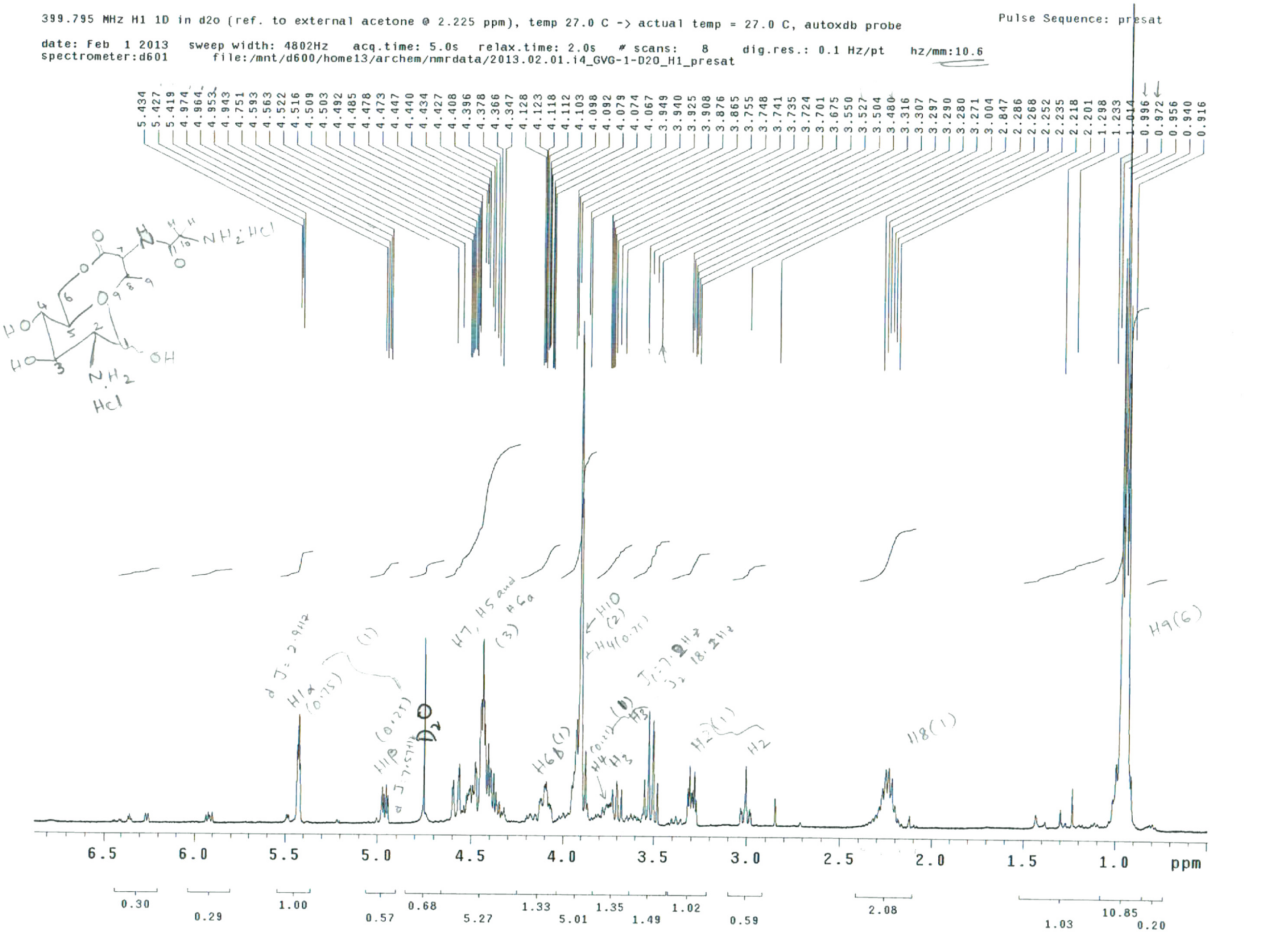
Characterization of the Gly-Val-COO-GlcN (GVG) ester derivative using 1H NMR spectroscopy. 1H NMR [CD3OD, 400 MHz]: [D2O, 400 MHz]: δ 5.40 (d, 1H, J = 2.9 Hz, H1α), 4.95 (d, 1H, J = 7.5 Hz, H1β), 4.30–4.65 (m, 3H, H5, H6a and H7), 4.06–4.14 (m, 1H, H6b), 3.90 (s, 2H, H10), 3.66–3.85 (m, 1H, H4), 3.45–3.65(m, 1H, H3). 3.00 and 3.30 (m, 1H, H2), 2.20–2.40 (m, 1H, H8), 0.99 (d, 6H, J = 8.6 Hz, H9).

**Fig 5:**
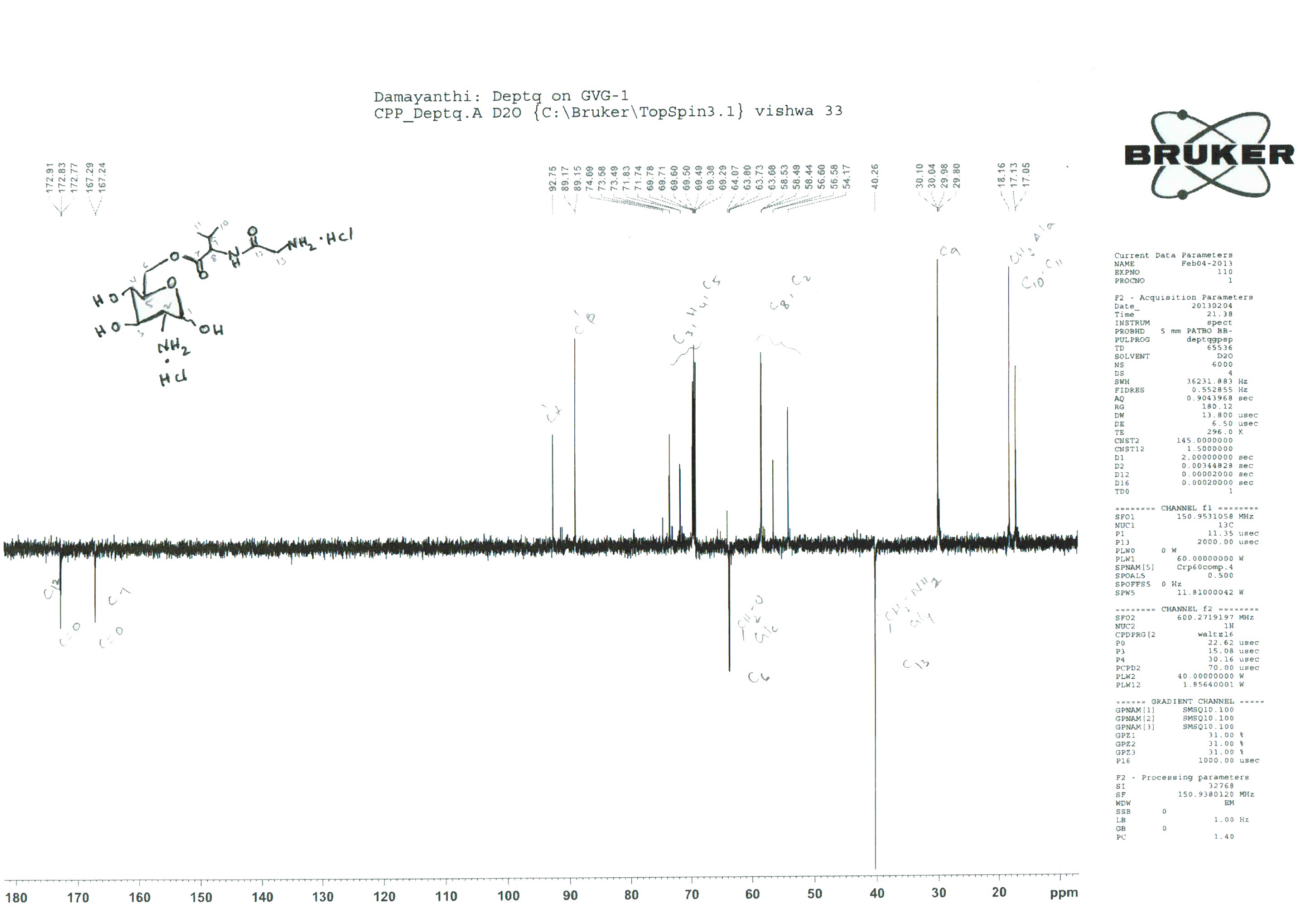
Characterization of the Gly-Val-COO-GlcN (GVG) ester derivative using 13C NMR spectroscopy. 13C NMR [D2O, 150 MHz]: δ 172.8 (C12), 167.2 (C7), 92.7 and 89.1 (C1α and C1β), 73.5, 71.7 and 69.5 (C3, C4 and C5), 63.8 (C6), 58.5 (C8), 56.5 (C2), 40.2 (C13), 30.0 (C9), 18.1 and 17.1 (C10 and C11).

GVG was further characterized using 1H and 13C NMR spectroscopy (Figs 4 and 5, respectively). The product GVG HCl exists as an inseparable mixture of α- and, β-isomers, approximately in the ratio of 1:3. Some protons of the same number, as shown in the structure (Fig 4), often appear at different ppm. The presence of isomers is the intrinsic property of the D-Glucosamine sugar moiety.

### Stability of GVG

Thermal stability of the GlcN derivative was evaluated by placing it in a 60°C oven for 48 h, as described under methods. In order to investigate the pH stability of the prodrug, it was incubated in an aqueous HCl solution (pH 2) and Krebs-Heneseleit bicarbonate buffer (pH 7.4) at 37°C for 2 hours. GVG showed acceptable thermal and chemical stability under the test conditions (>96% intact GVG).

### GVG in the presence of intestinal or liver homogenate

GVG was exposed to intestinal and liver homogenate in order to evaluate its stability as described under experimental section. GVG hydrolyzed to yield the parent drug, GlcN, in intestinal homogenate. The first order degradation rate constants and stability half-lives of the dipeptide-GlcN derivative in the rat intestinal homogenate were calculated as 0.04±0.008 min^-1^ and 17.9±3.8 min respectively. Analysis of the sample after incubation of GlcN prodrug with liver homogenate revealed that the prodrug was rapidly cleaved to the parent drug (GlcN) in less than 15 minutes. The parent drug, GlcN, was stable in the intestine and liver homogenates and did not degrade during the experiment period; the average recovery rate for GlcN was 98.3±1.3% after 60 min.

### Permeability study through everted rat jejunum sacks

**Fig 6:**
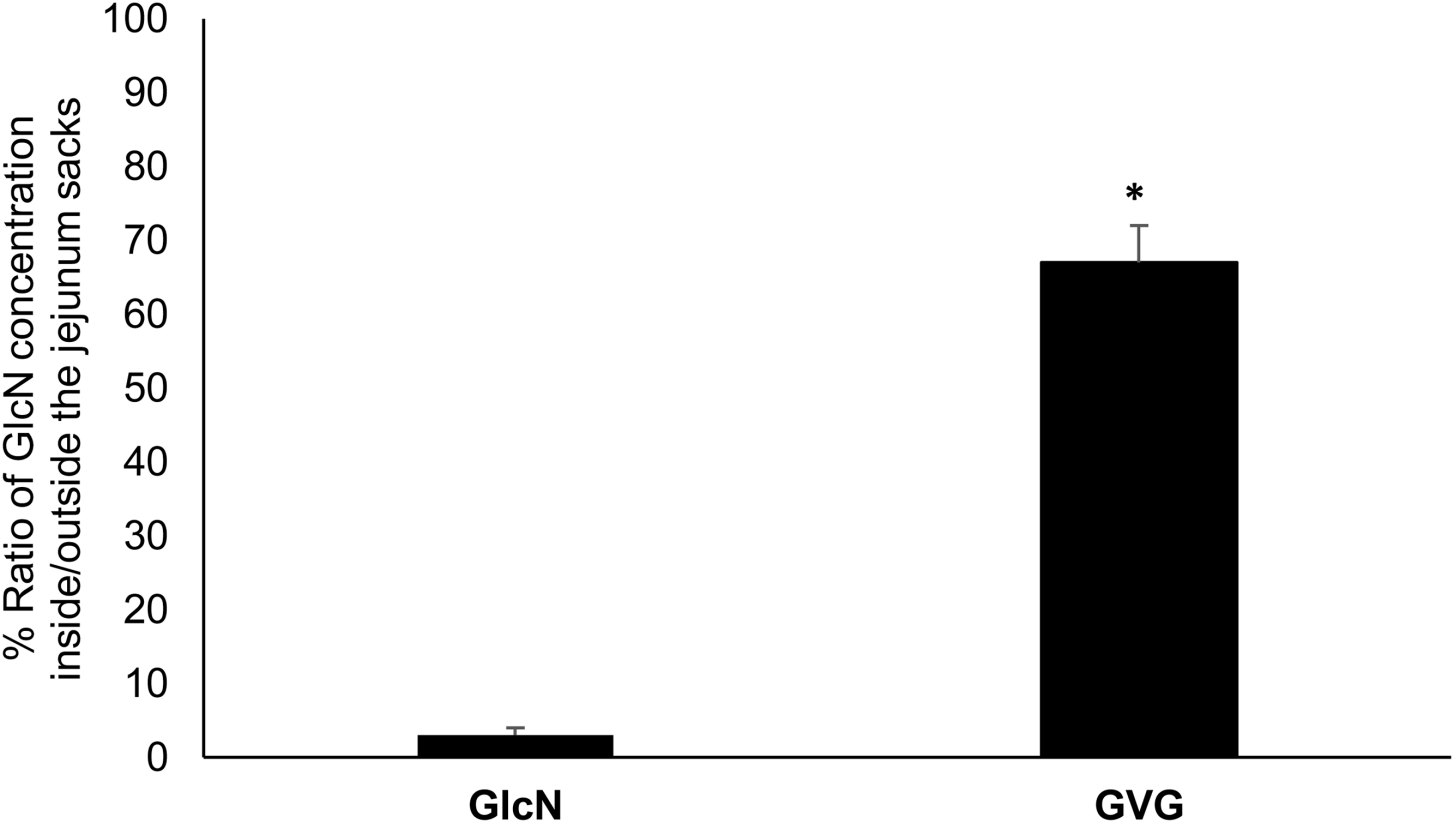
Percent average ratio of GlcN concentration (+SD) inside/outside the jejunum sacks (*n* = 6) after 60 min incubation with GVG or GlcN. * GVG significantly increased the ratio as compared to GlcN.

The GlcN prodrug was assessed for its permeability through rats’ jejunum sacks as described under experimental section. The evertet rat jejunum sac is a well-established method for *in vitro* permeation studies and it has been showed to maintain its tissue viability up to 120 min [32,33]. GVG significantly increased the gut permeability of GlcN inside the jejunum sacks. As depicted in Fig 6, the percent ratio of GlcN concentration inside/outside the jejunum sacks is significantly higher for GVG as compared to GlcN, at the end of the incubation experiments (t = 60 min). Additionally, GVG showed a significant increase in the cumulative amount of GlcN inside the jejunum sacks (serosal site) compared to GlcN at different time points (Table 1).

**Table 1:** Cumulative amount of GlcN (μg) inside the jejunum sacks (serosal site) (*n* = 6) at different time points, after incubation with 1.5 μM of GlcN or GVG.

Treatment	Time(min)			
	0	15	30	60
	Cumulative amount of GlcN (μg)			
GlcN	0	11.7±0.7^a^	25.8±1.1^c^	49.6±2.0^e^
Gly-Val-COO-GlcN (GVG)	0	80.1±10.8^b^	197.0±7.8^d^	358.8±14.1^f^

Data are shown as mean (±SD). Different superscript letters denote significant differences between means in a column (*P* < 0.05).

**Fig 7:**
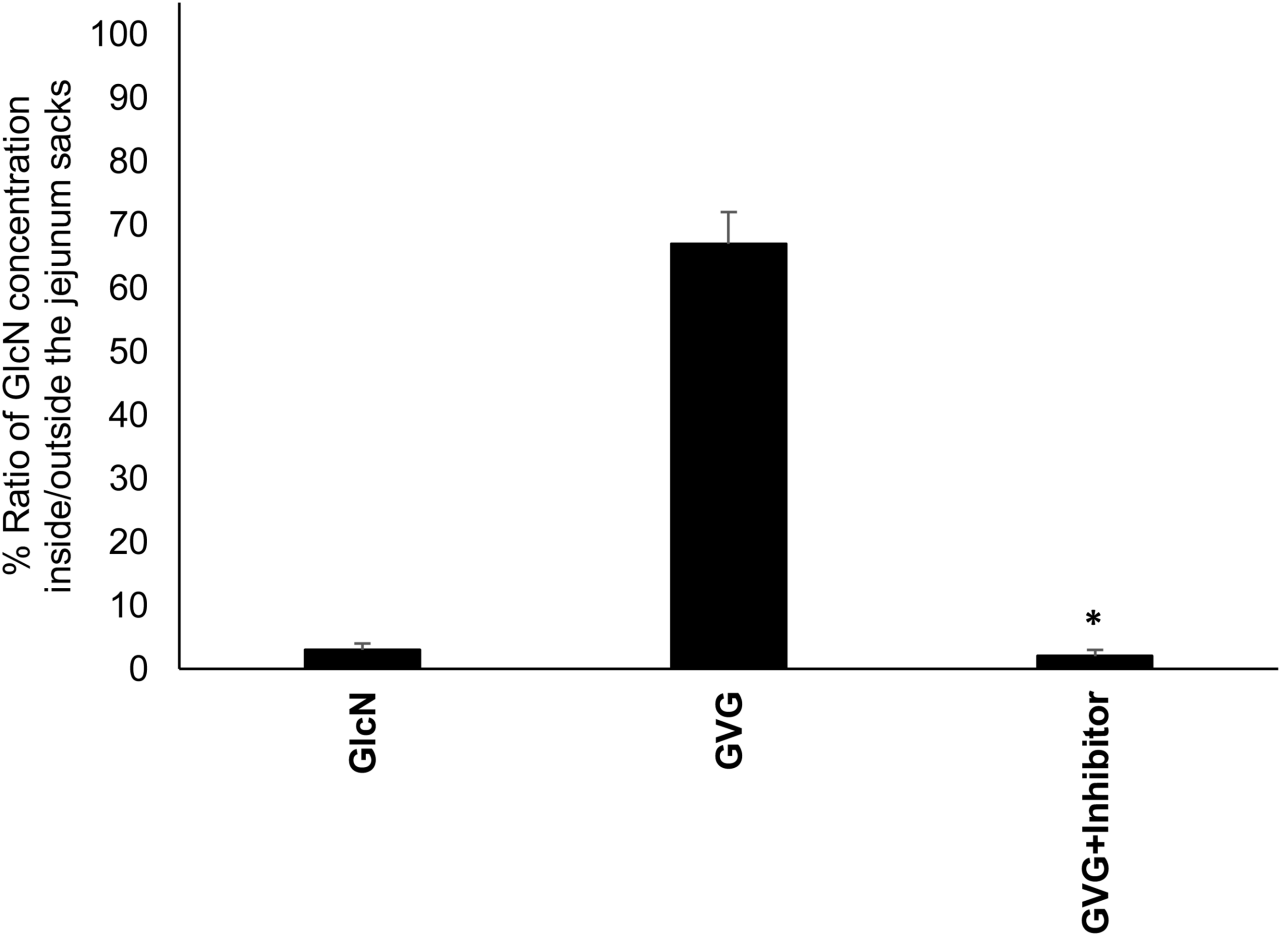
Percent average ratio of GlcN concentration (+SD) inside/outside the jejunum sacks after 60 min incubation with GlcN and GVG in the presence and absence of Gly-Sar (Inhibitor). *GVG permeability was significantly inhibited in the presence of Gly-Sar (*P* <0.05).

As detailed above, GVG showed a significant increase in both the cumulative amount of GlcN inside the jejunum sacks and the percent ratio of GlcN concentration inside/outside the jejunum sacks at the end of the experiment, which indicates an enhanced permeability across the gut membrane compared to GlcN. To examine if the increased permeability of GVG is due to its translocation by *PepT1*, permeability was measured in the presence and the absence of the *PepT1* specific substrate, Gly-Sar. GVG significantly increased the concentration of GlcN inside the jejunum sacks (serosal site), which in the presence of Gly-Sar, this increase was completely inhibited (Fig 7).

## Discussion

GlcN is a putative disease modifying agent [1] with mild anti-inflammatory properties [2,3]. Several animal studies have shown that GlcN can control inflammatory diseases such as adjuvant arthritis [2,3,20,21]; however, its low bioavailability limits its beneficial therapeutic effects [5]. Designing prodrugs to target a specific receptor in the gastrointestinal (GI) track in order to enhance bioavailability is one the widely utilized method in drug development [11]. The prodrugs are usually inactive entities that quickly yield the parent drug as soon as they enter the systemic circulation. Among several intestinal transporters, *peptide transporter 1 (PepT1)* has attracted a great deal of attention in recent years [11,12]. *PepT1* shows broad substrate specificity and transports di- and tri-peptides. Introducing an amino acid or di-peptide moiety into the molecular structure of parent drugs via an ester or amide bond is a common strategy in this matter [13,14]. New drug conjugates should have a desired physicochemical and intestinal stability. On the other hand they should convert to their parent drug as soon as they cross the GI membrane and enter the systemic circulation. The site of prodrug cleavage might be the plasma or the liver.

For the first time, we synthesized and characterized a Glycine-Valine ester conjugate of GlcN (GVG) with enhanced gut permeability. This is especially important given that GlcN is a highly water soluble compound and this study utilized a novel approach toward synthesizing peptide prodrug of a highly hydrophilic molecule. The ester derivative was synthesized using a solid-phase approach that is convenient, allows fast synthesis, and good yields of the purified product. In the classic solid-phase peptide synthesis, the N-protected C-terminal amino-acid residue is anchored via its carboxyl group to a resin [22]. However the synthesis of the ester derivatives of GlcN required a resin with the ability to anchor the amine group. 2-Chlorotrityl chloride resin [23] is a versatile acid labile resin that can be successfully used for the immobilization of amine or carboxylic groups. Cleavage of the functional groups is generally achieved using 1–50% TFA in DCM [23,24]. Herein, to the best of our knowledge for the first time, we synthesized a GlcN ester derivative with >98% purity and 67.8% yield by anchoring the amino sugar (GlcN) to 2-chlorotrityl chloride resin through its amine group and achieved the synthesis by using the stepwise solid phase synthesis on the resin.

One of our primary goals of this study was to synthesize a GlcN derivative with desirable physical stability. GlcN is available in the market as HCl and sulphate salts with the latter being the salt in the most of the products reported to have favorable clinical outcomes [25–27,34,35]. The bioequivalence and possible identical efficacy of the two salts has been discussed elsewhere [5]; however, since the sulfate salt is known to lack physical stability (unless formulated as crystalline GlcN sulfate with additional stabilizing salts) [7], we aimed to synthesize new GlcN derivatives with enhanced chemical stability. The stable derivatives do not require the addition of other stabilizing salts such as NaCl. This approach, in turn, provided the opportunity to reduce the size of the administrable bulk. A reduction in the size of the finished product is of primary importance; particularly for the elderly patients. The currently available data regarding the stability of the HCl salt is not conclusive; however the stability of the finished products can be an issue since GlcN HCl crystals are hygroscopic. Further investigations are needed in order to assess the stability of the HCl salt. Newly synthesized compound was tested for its physicochemical stability. GVG appears to have a desirable chemical stability. Next, pH stability of the newly synthesized compound was studied at two different pH’s, 2 and 7.4, which are corresponding with the normal pH of different segments of the gastro-intestinal track. GVG exhibited a high stability in the aforementioned pH conditions.

In addition, the prodrug was investigated for its GI stability, its ability to be biotransformed into the parent drug in the liver, and its transportability through the everted rat gut. GI stability is another prerequisite for suitable orally administered prodrug candidates, for if they cleave before reaching their site of absorption, they will lose their property to enhance the bioavailability of the parent drug [13]. GVG demonstrated cleavage half-lives of around 17 min in the intestinal homogenate. In another study [13], a series of acyclovir di-peptide prodrugs were synthesized and tested *in vitro* and *in vivo*. According to the authors, intestinal homogenate hydrolysis studies revealed that a minimum cleavage half-life of 15 min was desirable in order to have a potential candidate with increased bioavailability. Hence, GVG met this requirement. The ester prodrug was rapidly cleaved to yield GlcN after incubation with liver homogenates in less than 15 min, which is a desirable property of a prodrug.

In order to improve bioavailability, in addition to an acceptable stability profile, the GlcN prodrug needed to demonstrate an acceptable transportability via *PepT1*. Previously, the affinity of several di-peptides toward *PepT1* has been studied [28]. Many of the studied di-peptide sequences, exhibited high affinity toward the transporter, however high affinity may not always be translated into high transporter-mediated transportation after oral administration because the substrates might only bind to the transporter without being translocated [13]. We utilized the *in vitro* permeability test through the everted rat gut in order to determine the transportability of the GlcN derivatives. GVG fulfilled this criterion and showed a significant increase in the cumulative amount of GlcN inside the jejunum sacks. Likewise, Anand et al. showed that after oral administration, Gly-Val dipeptide ester prodrug of acyclovir appeared to be more efficiently absorbed across the intestinal mucosa compared to other ester derivatives [13]. We assumed that the increase in the permeability of the GVG prodrug was possibly due to the recognition of the conjugate by the *PepT1*. This is most likely due to the fact that this conjugate met the general requirements concerning the structures of the *PepT1* substrates, including possession of a di- or tripeptide skeleton with an appropriate three dimensional conformation, high affinity toward the transporter, and an appropriate small size [28].

As mentioned above, the increase in the permeability of GVG was possibly due to the recognition of the conjugate by the *PepT1*. To prove this assumption, we performed a permeability inhibition test with Gly-Sar, a specific *PepT1* substrate; Gly-Sar inhibited transport of GVG, indicating its penetration was through the *PepT1*.

## Conclusion

The GlcN di-peptide conjugate, Gly-Val-COO-GlcN (GVG), demonstrated a desirable chemical and physical stability and favorable gut permeability as compared to GlcN. The uptake of this conjugate was efficiently mediated by *PepT1*, as it was significantly inhibited in the presence of Gly-Sar. We, therefore, suggest GVG to be a desired GlcN prodrug in order to increase GlcN bioavailability. However, in addition to comparative PK studies, clinical trials are necessary to investigate the therapeutic outcomes of GVG as compared to the clinical outcomes of the regulated products of GlcN sulfate.
